# Middle-Aged and Young People's Perspectives on Healthy Aging Through Exercise: Environmental, Psychosocial, and Individual Factors With the Photovoice Method

**DOI:** 10.1155/jare/4578688

**Published:** 2024-12-23

**Authors:** Aysu Önal, Gökhan Deliceoğlu

**Affiliations:** ^1^Department of Training and Movement Sciences, Institute of Health Sciences, University of Gazi, Ankara, Turkey; ^2^Department of Coaching Education, Faculty of Sports Sciences, University of Gazi, Ankara, Turkey

**Keywords:** active life, aging, digital photographs, photovoice, physical activity

## Abstract

The aim of this study is to examine the individual's perspective on healthy aging through exercise. Individuals aged 18–60 years who have been physically active for at least one year were included in this study. The study focused on the exercise behaviors of young and middle-aged individuals through photographs. The photovoice method was used to discover how healthy aging affects exercise behaviors in physically active individuals. Content analysis was used to analyze photographs and the photovoice of the participants. Environmental, psychosocial, and individual themes emerged with the interpretation of the data. According to the results of this study, environmental, psychosocial, and individual factors have been found to be effective for healthy aging. The contribution of exercise to personal development was beneficial in strengthening the network of interpersonal relationships and improving physical and mental health in young and middle-aged adults. Exercise programs should not be monotonous, and individuals should not depend on a single location for exercise.

## 1. Introduction

The concept of healthy aging to improve quality of life has been widely researched [1]. Studies on healthy aging have become increasingly important in the last 20 years. Developing a framework for understanding what it means to age well may make it easier to guide people to healthy aging. Common terms have been studied in the field of aging (e.g., active aging and successful aging), and it has been emphasized that the most appropriate term is healthy aging [2]. This is because the World Health Organization defines healthy aging as “the process of developing and maintaining functional ability that ensures well-being at an advanced age” [3]. Researchers have noted that individuals experience a decrease in their functional capacities with aging but focus on minimizing losses to maintain levels of functionality and well-being [4]. Although many factors—including physiological, psychological, social, lifestyle, age, and gender—affect healthy aging [5–8], declines in functional capacity can be reduced with early intervention before the age of 50 [9]. The seven factors that predict healthy aging are not smoking, adaptive coping style, absence of alcohol abuse, maintaining a healthy weight, having a stable marriage, obtaining more years of education, and engaging in regular exercise [9].

Because exercise has the potential to facilitate behavior change [10], research on healthy aging is critically related to understanding lifestyle behaviors that require active participation, such as exercise [11, 12]. A decrease in functional limitations and disability risk has been observed in elderly people who participate in regular aerobic activities [13]. Physical activity has been associated with reduced all-cause mortality rates and an increased likelihood of living a longer and healthier life [14]. Moreover, it may be possible to maintain well-being through physical activities throughout one's entire life [15]. However, there is a gap in the literature regarding young people's approaches to aging and old age through exercise. It is important for young adults to assess their own aging to improve their perspectives and attitudes toward aging and to take better care of their current selves [16].

Understanding young people's perspectives and actions regarding healthy aging is an important indicator of well-being in old age in terms of how they impact older people in their societies [17]. However, the active involvement of young people in these processes does not eliminate the need to focus on the contributions and experiences of older adults [17]. For all these reasons, it is significant to investigate the reasons individuals participate in lifelong health-enhancing practices such as exercise. This study examined the perspectives of middle-aged and young adults on exercise and healthy aging.

## 2. Materials and Methods

### 2.1. Study Design

This study used a qualitative method including participatory action research. A total of five online meetings were held with participants and the research team, with one week between each meeting. This article was completed in 5 weeks. Participants engaged in discussions about exercise-related aging and were asked to reflect on questions about their own aging, answering them with photographs. These photographs were then analyzed together with the research team. This study was approved by the ethics committee of the University of Gazi (ID:2023-122) and adhered to the Declaration of Helsinki throughout the investigation.

### 2.2. Participants

Participants who had attended physical activity courses for at least one year were reached by email. Volunteers between the ages of 18 and 60 were included in the study. There were two groups: young adults aged 18–34 and middle-aged adults aged 35–60 (Table 1). Based on previous longitudinal and review studies involving middle-aged adults, we define middle age as between 35 and 60 years old [18–21]. Participants were enrolled in either the young or middle-age group according to their birth years. Additionally, all participants were required to have a smartphone or device capable of taking photographs. A total of 36 volunteers who met the criteria participated in the study. Participants were informed about the study by the researchers, and all signed the consent information form.

### 2.3. Procedure: Photovoice Method

The group nature of photovoice creates a strong foundation on which collaborators can encode data, create themes, and discuss and improve their experiences [22, 23]. Various photovoice methods are used in different populations for health promotion [24]. In this study, the photovoice method was used to discover how healthy aging affects exercise behaviors in physically active individuals. The photovoice method was explained to the participants by the research team at the first meeting. The photovoice procedure in this study was as follows [25, 26].

1. Formation of the group: Those who met the inclusion criteria were invited to the online meeting. During this session, the participants met each other and the research team.
2. Defining the theme together with the participants: Exercise was discussed in this context to determine whether it has an effect on healthy aging. Is there a relationship between exercise and healthy aging?
3. Taking photographs: Participants were encouraged to take photographs using their smartphones according to the guidelines presented at the end of these sessions. The guidelines: Participants were instructed to take at least 3 and at most 5 photographs reflecting healthy aging and exercise. Each participant was asked to take photographs on their own. Participants were allowed to show their creativity during the photo shoot.
4. Photograph selection and contextualization: The participants sent the photographs they took to the research team. All participants were asked to explain the photographs they sent in a few sentences. They were also asked to share additional thoughts reflecting on their photographs at online meetings. Participants were given enough time to talk in online meetings (∼10 min). The questions were asked to the participants in sequence. Why have you taken this photograph? What do you see here? How does this relate to healthy aging and exercise?
5. Targeting nongroup decision makers: At this stage, academics working on healthy aging and exercise were reached by email. Decision makers were informed about the issue, and photographs taken by participants at the meeting were shown to stakeholders (academicians, high school principals, and employees of the Ministry of Youth and Sports). Feedback from stakeholders on the issue was received. It was aimed to spread awareness of healthy aging and exercise with the participation of stakeholders.

### 2.4. Data Collection and Analyses

A conventional content analysis was used to analyze photographs and the photovoice of the participants. Content analysis was carried out following the processes of data collection, coding, development of categories and themes, and identification and interpretation of findings [27, 28]. The themes were revealed through semistructured online interviews with open-ended questions in the photovoice method used in the study. Interviews continued using the purposeful sampling method until data saturation was reached [29]. The questions were asked to better explain the details. All conversations were recorded and transcribed. The text was read by the researchers several times, and statements unrelated to the subject were removed. Then the sentences related to the main meaning were divided into semantic units. Appropriate codes were written for each meaning unit. The codes that did not reflect the opinions of the participants were corrected. Categories were created from codes with similar meanings. According to the level of interest of the category, subthemes and the general theme of the subthemes were created. The research team reached out via email to community stakeholders working on healthy aging and exercise. To reduce research bias, the researchers shared the photovoice study with experts in the field and invited them to clarify, elaborate, and criticize the comments in individual meetings. In addition, the cooperation with stakeholders, feedback, and comments was done to strengthen the reliability of this qualitative study [30].

## 3. Results

The demographic characteristics of the participants are shown in Table 1. In the photos examined by participants and researchers, three main themes were identified in both the young and middle-age groups as impacts of exercise related to healthy aging factors. These themes were environmental, psychosocial, and individual factors. A subtheme was determined for each main theme.

### 3.1. Young Group

#### 3.1.1. Theme 1: Environmental, Subtheme: Outdoor and Indoor Exercise

**Figure 1:**
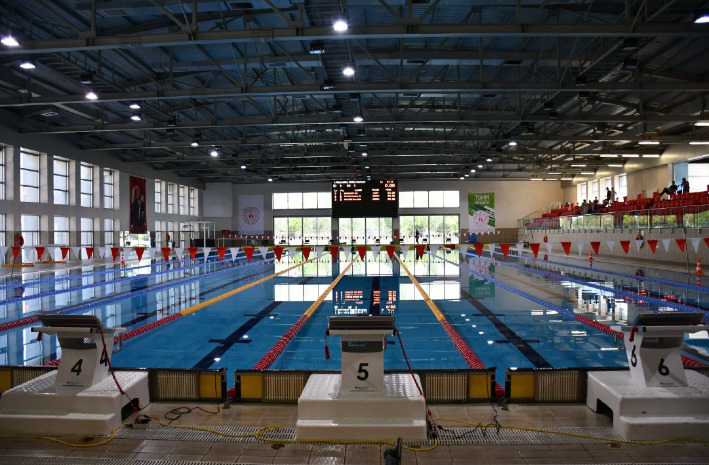
Environmental factors for young groups.

The young participants expressed that they enjoyed going to natural environments where they could exercise comfortably for healthy aging. Additionally, they stated that although they prefer clean and natural environments, they may also choose indoor spaces for the continuity of their training (Figure 1). The participants mentioned that having a variety of equipment available while exercising and the presence of an expert trainer when necessary are important for maintaining their training routine.

#### 3.1.2. Theme 2: Psychosocial, Subtheme: Positive Emotions

In this theme, the young participants explained that exercise helps them feel happy and energetic. They mentioned that, thanks to exercise, their negative feelings decreased while positive feelings such as relaxation, feeling alive, and well-being increased. The participants expressed their belief in more successful aging through increased positive emotions. “Happy people age happily” (P1). They emphasized that having good mental health is extremely important at any age.

#### 3.1.3. Theme 3: Individual, Subtheme: Physical Image

**Figure 2:**
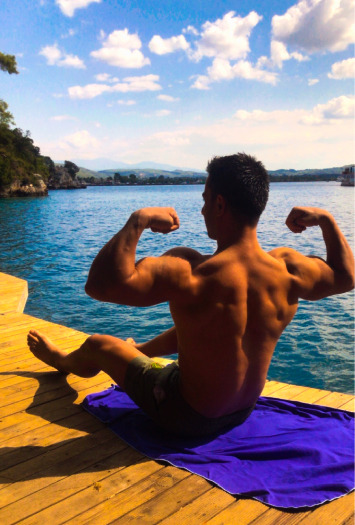
Individual factors for young groups.

The individual reason the young group cares about healthy aging is their physical appearance. The participants showed the muscularity of their bodies as evidence of healthy aging. “I believe that if I improve my body, I will age healthier in my later years” (P2). Participants said that the most important reason to start exercising is visual appeal. They chose exercise as a hobby due to the desire to be liked by both themselves and others (Figure 2).

### 3.2. Middle-Aged Group

#### 3.2.1. Theme 1: Environmental, Subtheme: Indoor Exercise

**Figure 3:**
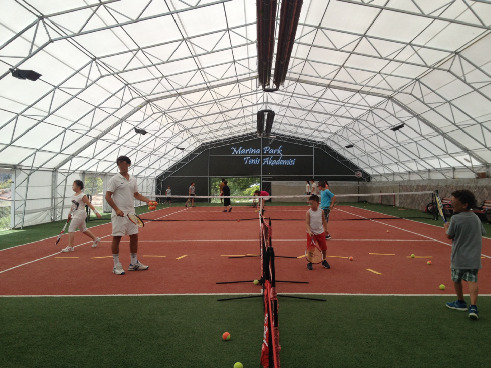
Environmental factors for middle-aged groups.

The participants explained that exercising in a safe and accessible area is important for the continuity of the training. “I lose some of my time when I go to remote places to play sports, instead I can devote more time to sports by going to nearby places” (P3). Participants also stated that they preferred safe places where there were facilities such as light, heat, toilet, shower, and a club/academy where member entry and exit control was carried out, as an exercise area (Figure 3). They explained that the reason for these preferences is to be able to make training continuous by enjoying the exercise more. The participants emphasized that exercise should be done constantly for healthy aging.

#### 3.2.2. Theme 2: Psychosocial, Subtheme: Sociability

**Figure 4:**
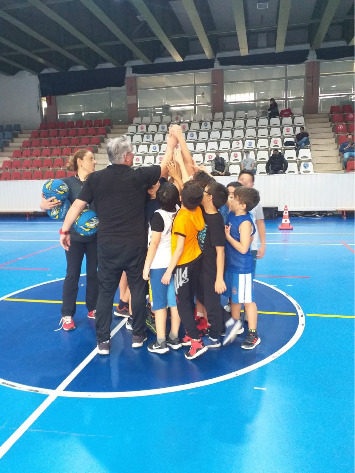
Psychosocial factors for middle-aged groups.

Middle-aged people said that socialization is necessary for successful aging and emphasized that exercise activities are the most basic means of socialization. Individuals expressed that they felt that they considered themselves a part of society when they socialized. Thanks to the exercise, they expressed that they had passed on their own experiences by coming together not only with their peers but also with individuals in other generations (Figure 4). “Interpersonal communication is not limited to casual conversations; talking about useful topics like exercise allows me to make connections with other individuals” (P21). Therefore, the participants told them that they felt more happy.

#### 3.2.3. Theme 3: Individual, Subtheme: Be Healthy

In this theme, the participants clearly stated the importance of staying healthy first to be able to age healthily. The middle-aged participants stated that the easiest and cheapest way to stay healthy is exercise. “As I have learned more about the various health benefits of being physically active, exercise has reached a point in my life that I cannot give up” (P4). “For me, being healthy means walking without fatigue” (P25).

## 4. Discussion

In this study, the perspective of exercise associated with healthy aging was examined among adult individuals. According to the results of this study, environmental, psychosocial, and individual factors are significant in promoting healthy aging through exercise. This underscores that physical activities encompass a combination of various elements [31]. The more important the meanings were, the more likely their participants were to be physically active [31].

Over the past 15 years, there has been increasing interest in the relationship between exercise and health performed in outdoor environments. Walking in natural environments is known to have more positive effects on health compared to urban environments [32, 33]. Despite these benefits, some groups prefer indoor exercise. The findings obtained in middle-aged participants align with studies highlighting the importance of safety and comfort in influencing physical activity levels [34, 35]. In contrast, for young adults in our study, exercise place was less critical than the sustainability of their exercise routines, reflecting emotional connections and behavioral determinants for maintaining physical activity [36].

Psychosocial conditions also significantly influence physical activity levels. Current evidence shows that increasing physical activity is associated with reduced negative mental states [37, 38]. Both young and middle-aged adults reported physiological and psychological relaxation through physical activity [32, 39, 40]. Moreover, high-intensity aerobic exercises and moderate-intensity aerobic exercises have been linked to improved mood [41, 42], with potential antidepressant effects attributed to neurotransmitter activity and neurotrophic factors like brain-derived neurotrophic factor (BDNF) [43, 44]. Therefore, comprehensive strategies are essential to promote well-being and positive aging despite challenges in later life [5].

Regular physical activity offers extensive health benefits for older adults [45, 46], yet many do not meet recommended activity levels [47]. Common barriers cited by adults include fatigue, time constraints, and inadequate program suitability [21]. Physical strength, such as muscle tone and fitness, is valued across all age groups for its individual health benefits [21, 48]. In this study, middle-aged adults preferred to exercise to benefit from health benefits rather than to look physically muscular. This perspective can help delay, prevent, or manage the costly and challenging chronic disease faced by older adults [49].

Because of the many benefits of exercise on preserving functional capacity, fostering societal integration, and reducing ageism, people thought that they delayed the effects of aging when they participated in physical activity. Understanding the physical activity needs of different age groups can inform coaches and recreation leaders in designing effective programs. Nonetheless, this study has revealed that healthy aging is valuable for every age group.

## 5. Conclusions

To promote successful aging, it is crucial to develop lifelong physical activity programs tailored to individual interests and capabilities, guided by expert coaches. Programs should encompass diverse activities and venues to sustain long-term engagement. Facilities should be designed to meet local physical activity needs to ensure sustained use. In the early stages of creating a community-based exercise habit, the focus should be on interesting physical activities to increase participation in exercise.

### 5.1. Limitation and Future Research

This study focused on healthy, physically active individuals, and exercise approaches may differ among those with chronic diseases. Future research should explore exercise perspectives among individuals with chronic conditions to better understand their needs. Additionally, future studies could investigate gender differences in exercise perceptions and explore how exercise relates to healthy aging among disadvantaged communities. Digital platforms should be leveraged to enhance community participation in physical activities, promoting social connection and reducing isolation and ageism. Reliable physical activity communities on digital platforms should be introduced to individuals, and increasing the number or functions of the communities should be encouraged. Thus, as people read the articles/blog of the participants in the activity or see their photographs in the activity, their interest in sports remains alive and individuals of all ages can find the opportunity to exercise with each other. We think that this study will help communities make their voices heard to improve their lives and guide them to actively participate in activities in accordance with the United Nations Decade of Healthy Aging (2018–2030) action plan [50]. In addition, in future studies, more awareness of healthy aging and exercise issues should be created through active participatory studies in which communities can freely express their thoughts.

## Figures and Tables

**Table 1:** Demographic characteristics of the participants.

Variables	Young	Middle-aged
N	20	16
Age (years)	23.0 ± 4.47	41.6 ± 5.92
Weight (kg)	67.17 ± 13.72	66.8 ± 13.72
Height (cm)	1.73 ± 0.08	1.70 ± 0.06
High school	18.8%	20%
University	81.3%	80%

## Data Availability

The raw data can be requested from the corresponding author.
